# Preferring Lateral Video Endoscopic Inguinal Lymph Node Dissection Over Classic Video Endoscopic Inguinal Lymph Node Dissection in Squamous Cell Carcinoma of Penis: Lessons Learnt from Twenty-One Patients at a Single Center

**DOI:** 10.5152/tud.2023.23097

**Published:** 2023-11-01

**Authors:** Pradhuman Yadav, Amit Sharma, Deepak Kumar Biswal, Raghavendra RT

**Affiliations:** Department of Urology, All India Institute of Medical Sciences, Raipur, India

**Keywords:** Lymphadenectomy, inguinal, endoscopic, penile, squamous cell carcinoma

## Abstract

**Objective:**

Inguinal lymphadenectomy is essential for staging and disease control. Minimally invasive techniques are recently replacing open techniques to reduce complications. We present our experience and lessons learnt from 21 patients who underwent lateral video endoscopic inguinal lymphadenectomy (L-VEIL) for penile malignancy.

**Methods:**

All patients above 18 years of age with histopathology-confirmed squamous cell carcinoma penis with stages ≥ T1b and T1a with persistent lymphadenopathy who underwent L-VEIL over a period of 2 years (2020-2022) were included. The data were analyzed on the basis of intraoperative and postoperative complications, lymph node yield, hospital stay, and histopathology report.

**Results:**

Forty-one lower limbs of 21 patients underwent L-VEIL during the abovementioned period. Median age was 52 years. Mean operative time (on 1 side) was 80 minutes. Median lymph node yield per side was 7.2. Intraoperatively, 1 patient had a vascular injury at the saphenofemoral junction, requiring conversion to open. Postoperative complications were superficial surgical site infection (n = 4), lymphedema (n = 1), and lymphocoele (n = 3), one of which was drained by pigtail catheter. One patient required exploration on the second postoperative day because of vascular injury. Average duration of hospital stay was 3 days. The median time of drain removal was 13 days. Histopathology suggested seminoma in 1 patient and mature teratoma in 1 patient; the rest of the patients’ reports were negative for malignancy.

**Conclusion:**

The L-VEIL is safe and feasible, and there is a reduction (~30%) in complications; oncological outcomes are also not affected. It has better ergonomics, resulting in ease and comfort for surgeons when compared with classical VEIL.

**Main Points**

- Inguinal lymphadenectomy is an integral part in the management of patients with penile squamous cell carcinoma to provide improved outcomes.
- Open inguinal lymphadenectomy has higher rates of cutaneous and lymphatic complications.
- Minimally invasive techniques have fewer postoperative complications, but the intraoperative times are longer with minimally invasive techniques.
- Video endoscopic inguinal lymphadenectomy (VEIL) is safe, feasible, and has fewer cutaneous, surgical site, and lymphatic complications and similar lymph node yield, thereby providing similar oncological outcomes.
- Lateral VEIL is better in terms of intraoperative blood loss and surgeons’ comfort than classic VEIL.

## Introduction

Penile squamous cell carcinoma (SCC) is a common malignancy among elderly men in their late (fifth to seventh) decades. The most common sites for metastases are inguinal lymph nodes, and these are considered an important prognostic indicator for survival.^1-4^ About 30%-50% patients present with palpable nodes, and 10%-30% of patients with no palpable nodes have also been reported to have microscopic metastasis.^1-5^ This suggests that lymphovascular invasion occurs early, and hence, the management of inguinal nodes cannot be overemphasized for survival of such patients.

Inguinal lymphadenectomy (IL) was traditionally done by open method; but, this procedure has its own complications.^6^ It has high morbidity, with skin necrosis and postoperative wound infection being the main causes of morbidity. Lymphatic complications like lower limb edema, lymphocele, and lymphedema are also common.^6^

In order to decrease these complications, several modifications to the open procedure were suggested and practiced.^6^ Some of these procedures are skin-sparing techniques, sartorius muscle transposition, saphenous vein preservation, superficial and modified inguinal lymph node dissection, dynamic sentinel lymph node biopsy, and use of thick skin flaps.^6^

In this era of advances in minimally invasive techniques, minimally invasive approaches have also been tried. Laparoscopic/robot-assisted video endoscopic IL (VEIL) has gained popularity in terms of reduction in complications as well as similar oncological outcomes. This VEIL technique is a new technique described first by Tobias-Machado et al^7^ in 2006. Instead of using inguinal incision for lymph node dissection, they used 3 ports and created a plane deep to Scarpa’s fascia and infused gas to proceed with inguinal lymph node dissection in the same way as it is done in open surgery. They reported no complications with VEIL, and the patient had no disease progression at 25 months of follow-up. After this report, VEIL started gaining popularity because of low complication rates and similar oncological outcomes.

With this background, we share our experience of VEIL in inguinal node-positive patients operated at our institute. Our objective is to assess the outcome of patients with penile malignancy who undergo VEIL for inguinal lymph nodes. We describe the points of techniques and outcomes of patients who underwent classic VEIL and lateral VEIL (L-VEIL) with respect to surgical time, intraoperative complications like vascular injury, lymph node yield, postoperative complications (cutaneous, hematoma, and lymph-related), duration of hospital stay, and final histopathology report.

## Material and Methods

This prospective study was conducted at a tertiary care center in Central India over a period of 2 years from April 2021 to March 2023. The study received approval from Institutional Ethics Committee of Department of Urology and Renal Transplant, All India Institute of Medical Sciences (AIIMSRPR/IEC/2022/1082), and written informed consent was taken from all the patients for participation in this study. All patients more than 18 years of age with histopathology-confirmed SCC penis with stage T1a with persistent lymphadenopathy, stages ≥ T1b with clinical N0 or palpable nodes were included in this study.

### Preoperative Preparation

All included patients were admitted and evaluated with routine blood investigations and optimized before surgery. All patients received preoperative antibiotics 1 hour before surgery.

### Surgical Procedure of Video Endoscopic Inguinal Lymphadenectomy

**Figure 1.**
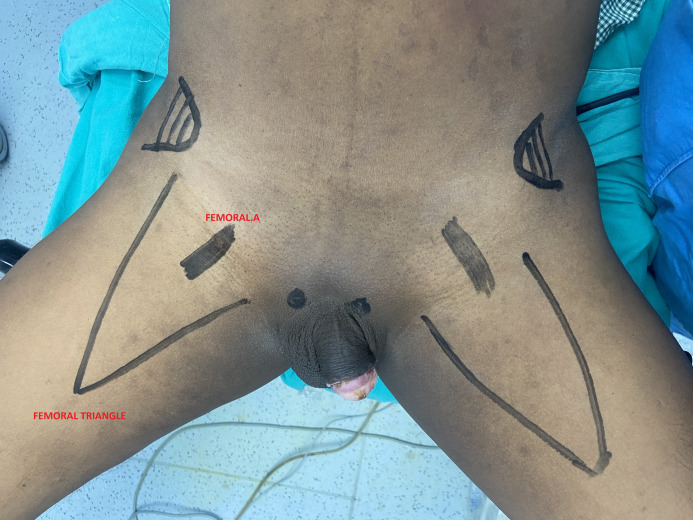
Surface marking of landmarks in video endoscopic inguinal lymphadenectomy.

The procedure was done under general anesthesia with the patient in supine position. The patient received intravenous third-generation cephalosporin antibiotic at the time of induction. Surface marking was done for femoral triangle, anterior superior iliac spine, inguinal ligament, femoral artery, and saphenofemoral junction (Figure 1). For classic VEIL, the patient’s lower limb was flexed at knee with external rotation of thigh and strapped to the operating table. The video monitor was placed at the opposite side. A 1.5 cm incision was given 2 cm distal to the apex of femoral triangle, and the plane was made deep to Scarpa’s fascia with 10 mm trocar. Thirty-degree camera was introduced through this incision, and CO_2_ was insufflated at 12-14 mmHg to create space. Two more ports, 10 mm and 5 mm, were inserted under vision. The 10 mm port was inserted 2 cm above and 6 cm medial to the camera port, and the 5 mm port was inserted laterally in symmetrical position.

**Figure 2.**
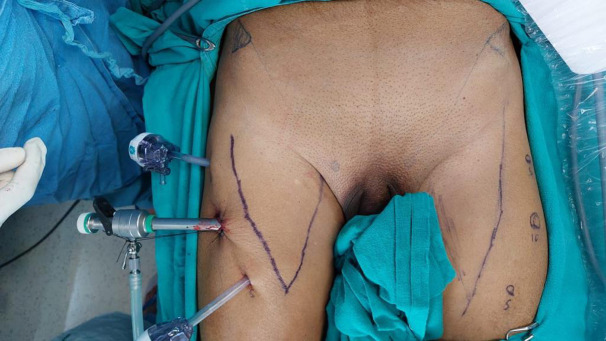
Port positioning in lateral video endoscopic inguinal lymphadenectomy.

The first port (10 mm) was placed 2 cm lateral to the lateral boundary of femoral triangle and deepened up to the fascia lata (Figure 2). Space was developed on either side with blunt and sharp dissection by finger; 2 working ports were placed by finger-guided technique on either side of the first incision. CO_2_ insufflation was done in the same way as for classical VEIL.

**Figure 3.**
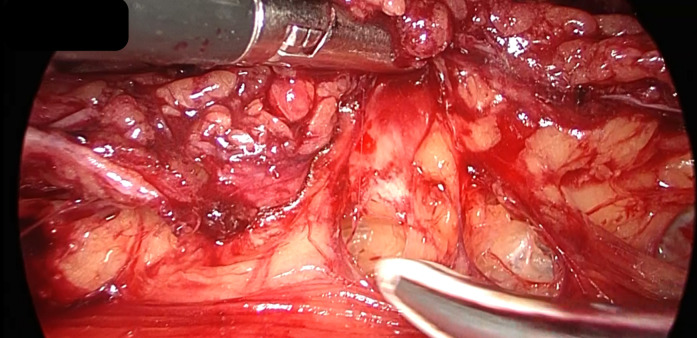
Intraoperative image depicting dissection in lateral video endoscopic inguinal lymphadenectomy.

**Figure 4.**
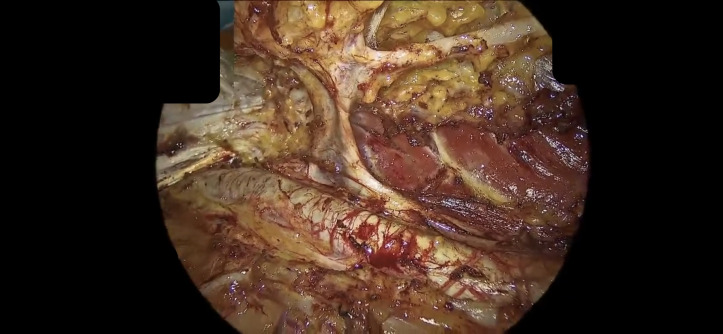
Intraoperative image showing the final image before the retrieval of specimen.

**Figure 5.**
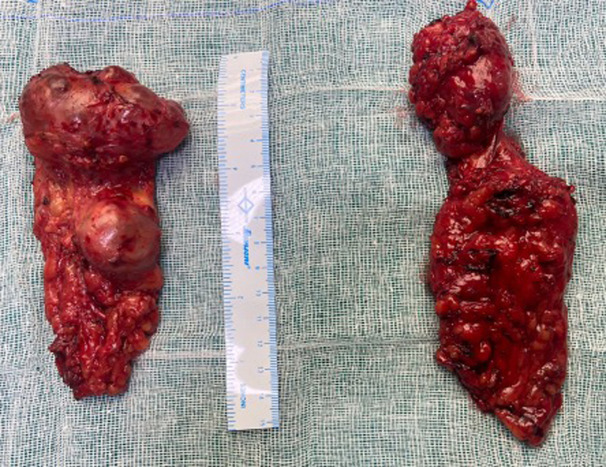
Image of the excised specimen in bilateral video endoscopic inguinal lymphadenectomy.

This was followed by doing both sharp and blunt dissection from medial to lateral side (Figure 3), and all fibrofatty tissue in femoral triangle was dissected from underlying femoral vessels. Saphenofemoral junction was then identified, clipped, and divided. The fibrofatty tissue from skin deep to Scarpa’s fascia was then dissected by taking care to avoid skin buttoning. All fibrofatty tissue was placed in retrieval bags and removed from 10 mm port. Figure 4 depicts the final image before retrieval of specimen, and Figure 5 depicts the excised bilateral VEIL specimen. The procedure ended with the placement of suction drain, removal of ports, and skin closure.

The data were collected and analyzed on the basis of surgical time and intraoperative complications like vascular injury, lymph node yield, postoperative complications (cutaneous, hematoma, and lymph-related), duration of hospital stay, and final histopathology report.

The collected data were then assessed for normality, and normal distribution data are reported as mean with SD, and skewed data are reported as median with range.

## Results

During the abovementioned period, 41 thighs of 21 patients underwent VEIL. Table 1 summarizes the demographic characteristics of the patients included in the study. Among the 21 patients, 4 had a history of tobacco chewing, 6 had a history of smoking, and 3 had a history of alcohol consumption. Five patients were diabetic, 6 were hypertensive, and 2 had ischemic heart disease. Five patients (2 bilateral and 1 unilateral making, n = 9) underwent classic VEIL, and 16 patients (n = 32, all bilateral) underwent L-VEIL. The single patient who underwent unilateral VEIL had already undergone unilateral open IL at another hospital. Thus, the analysis of data was done taking n as 41. Among these, 2 patients had T1a disease with bilateral palpable inguinal nodes, 9 had T1b disease, and 10 had disease with stage T2 or more.

Intraoperative events are summarized in Table 2. The median operative time (on 1 side) was 80 minutes (range 50-120 minutes). Mean lymph node yield per side was 7.2 (range 2-11). Intraoperatively, during classic VEIL, 1 patient had vascular injury at the saphenofemoral junction, requiring conversion to open procedure. Postoperative complications are summarized in Table 3. These were superficial surgical site infection in 4 patients (managed conservatively with local wound care and antibiotics), lymphedema in 1 patient (managed conservatively), and lymphocoele in 3 patients, which was drained by pigtail catheter in 1 patient. The other 2 patients with lymphocoele were managed conservatively. One patient required exploration on the second postoperative day after L-VEIL because of vascular injury. The median time of drain removal was 13 days (range 10-16 days). Median hospital stay was 3 days (range 2-4 days). Malignancy was detected in lymph nodes of 2 patients (seminoma in one and mature teratoma in the other); rest were reactive nodes.

At follow-up, 1 patient died of cardiac cause; the rest are on regular follow-up.

## Discussion

The importance of IL in patients with penile malignancy cannot be overemphasized as there is definite survival benefit. Conventional open IL has significant morbidity. Two-thirds of the patients have minor complications like superficial wound dehiscence, mild edema, and seroma formation, and about one-third of the patients suffer from major complications like flap necrosis, deep-vein thrombosis, and lymphocele which may require drainage.^6^ Consequently, there have been ongoing efforts to modify the operative procedure of open IL so as to minimize the complications. Taking smaller skin incisions, using thicker skin flaps with good blood supply, restricting dissection medially, preserving the saphenous vein, and transpositioning of artorius muscle were some of the techniques used during open IL to decrease the complications.^6^

However, approximately 36% minor complications like deep-vein thrombosis have been reported even with these modifications.^6,8,9^ In the era of minimally invasive surgery, efforts were made to develop such techniques for IL. Several investigators took efforts to develop techniques to perform less invasive endoscopic methods for IL.^10^ The VEIL technique was first described by Tobias-Machado et al,^7^ in 2006, in order to replicate the standard radical IL based on similar oncological outcomes and at the same time reducing morbidity and minimal complications.^6^

This new technique, VEIL, by Tobias-Machado created a lot of interest among the urologists and is being accepted for IL.^8,10^ Since its introduction in 2006, many centers have adopted this approach and reported favorable results with less complications and good outcomes.^10,11^ Sotelo et al^12^ reported lesser cutaneous complications with VEIL when compared with open approach.^10^ Solsona et al^13^ compared open and VEIL procedure and concluded that VEIL decreases postoperative morbidity without compromising oncological control.^10^ Pompeo et al^10^, in 2013, reported that simultaneous bilateral VEIL is also feasible, thereby reducing the anesthesia and operative time without compromising the oncological outcome.

In fact, Josephson et al,^14^ in 2009, and Dogra et al,^15^ in 2011, have reported good outcomes with robot-assisted VEIL.^6,14,15^ This suggests that even though VEIL is technically challenging and requires working in smaller space, the good results are reproducible even with robotic system and may even become accepted as the standard care of treatment for IL.^6,10^ Prophylactic inguinal lymph node dissection for urethral and vulval cancers may also be done by VEIL.^6^

Complications are less with VEIL when compared to open IL, and hence the postoperative morbidity is reduced in patients undergoing VEIL.^6^ Skin-related complications are decreased significantly.^6^ This facilitates early discharge and less financial burden on the poor patients. Correa et al^16^ and Thyavihally and Tongaonkar^17^ have reported minimal cutaneous and lymphatic complications in their studies. Other studies have also reported similar experience.^6,10,17^ In our study, superficial site infections (Clavin–Dindo grade 1) were observed in 4 patients.

In our study, there were 2 patients with lymphedema and 1 patient had lymphocoele, which required drainage. These findings are comparable to other studies. Tobias-Machado et al^18^ have reported only lymphorrhea and hematoma as complications. Wang et al^19^ have reported fewer surgical site-related complications with VEIL when compared with open procedures. Kumar et al^20^ reported a significantly lower wound complication rate and shorter hospital stay in VEIL patients when compared with open lymphadenectomy.^20^ However, lymphatic complications were similar for both the procedures.^20^

Chaudhary et al^6^ reported longer intraoperative time during VEIL procedure than with open IL.^6^ However, small incisions with better subcuticular closure gave a good esthetic outcome.^6^ Master et al^21^ reported comparable operating times between VEIL and open procedures and lesser morbidity with VEIL in their study on 25 patients. Tobias-Machado et al^7^ have reported a mean intraoperative time of 120 minutes and no intra- or postoperative complications with a mean lymph node yield of 8 in their study on single-site VEIL .^7^ We had an intraoperative time of 80 minutes in 1 thigh (range: 50-120 minutes).

In standard or classic VEIL, the camera port is placed in the longitudinal axis of thigh, and the 2 working ports are placed on either side of the camera port.^18,22^ In this classic VEIL, the surgeons face problems with respect to ergonomics. We operated on 5 patients initially by this technique and faced 2 problems. First, there was a restriction of camera movement by the patient’s thigh and second, the operating surgeon had to extend one hand over the thigh to maneuver the instrument placed in the opposite working port. Hence, we used L-VEIL technique in the next 16 patients, where we placed all the ports laterally along the long axis of the thigh. Consequently, the ergonomics improved, and it was less tiring for the operating surgeon to maneuver both the working instruments. Another advantage of L-VEIL was reduction in vessel injury, as direct visualization of saphenofemoral junction was possible. We operated 9 thighs in 5 patients with classic VEIL, and 1 amongst them required conversion to open because of vascular injury. We operated 32 thighs in 16 patients by L-VEIL, and 1 patient had to be reexplored on second postoperative day because of bleeding.

With respect to intraoperative complications, Nayak et al^22^ have reported more blood loss, mean nodal positivity, and mean hospital stay, lesser mean nodal yield, and increased drain output in patients who underwent open IL when compared to L-VEIL.^22^ However, they reported increased intraoperative time for L-VEIL.^22^ Similar results with significantly higher operative time with L-VEIL have been reported by Martin et al^23^, Wang et al^19^, and Tobias-Machado et al.^22,24^ However, the operative time with L-VEIL was reported to be lesser than the operative time reported with conventional VEIL in various studies.^10,11,22,25^

In our study, there was not much blood loss, and only 1 patient required blood transfusion—the one who suffered intraoperative vascular injury. This is similar to the study by Jain et al^26^ but lower than the blood loss reported by Wang et al^19^ with central VEIL.^22^ Thus, it is well apparent that blood loss is minimized by L-VEIL, and consequently the chances of postoperative infection are also reduced. The reason behind this reduction of blood loss is that the port site is away from the femoral blood vessel in L-VEIL. Closure with sartorius flap is also not required in L-VEIL.

In our study, the median nodal yield was 7.2 nodes per thigh. This is similar to that reported by Matin et al^27^(5-21 nodes on the left and 6-17 on the right side).^27^ In a study of 21 patients with gynecological malignancies, Li et al^28^ have reported the median lymph node retrieval to be 13, and all were negative to metastasis.^28^ Video endoscopic inguinal lymphadenectomy is definitely associated with decreased hospital stay as observed in our study. The median hospital stay was 3 days, which is similar to the reports in literature.^29^

Although, L-VEIL is technically challenging, it requires experience to work with ease in less space. It is safer and has lesser complications. The technique requires a few critical steps: distal lymphatic tissue division at the femoral triangle apex and proximal control of all lymphatic tissue at the deep portion of femoral channel using clips or tissue sealers.^30^^30^ Also, the lymph node yield and oncological outcome is also good.

There are limitations in our study—single-center experience, short follow-up, and fewer number of patients. However, more population-based randomized comparative trials with a larger sample size and longer duration of follow-up are required to establish the above conclusions. More comparative studies comparing classic VEIL and L-VEIL would also be required to establish the efficacy of one technique over the other.

## Figures and Tables

**Table 1.** Demographic Profile of Patients Who Underwent Video Endoscopic Inguinal Lymphadenectomy

S.NO.	Demographic Profile	No
1	Total number of patients	21
2	Total number of groins/thighs (units)	41
3	Classic VEIL (number of units)	9
4	Lateral VEIL (number of units)	32
5	Median age (years)	52 (range: 24-75)
6	Coorbidities:	13 (total)
	Diabetes	5
	Hypertension	6
	Ischemic heart disease	2
7	Disease stage:	–
	T1a	2
	T1b	9
	T2 or more	10

VEIL, video endoscopic inguinal lymphadenectomy.

**Table 2.** Summary of Intraoperative Events

S.No.	Intraoperative Events	
**1**	Median intraoperative time in minutes (per side)	80 (range: 50-120)
**2**	Median lymph node yield (per side)	7.2 (range: 2-11)
**3**	Vascular complication requiring conversion to open procedure (injury to SFJ [Safeno femoral Junction])	1
**4**	Reexploration required at second postoperative day because of slipping of vascular clip	1

**Table 3.** Summary of Postoperative Events

S.No.	Postoperative Events	No	Management
**1**	Cutaneous complications: SSI (Surgical Site Infection) (Clavien–Dindo grade 1)	4	Conservatively
**2**	Lymphatic complications		
	Lymphedema	1	Conservatively
	Lymphocoele	3	2 = Conservatively 1 = Pigtail catheter drainage
**3**	Mean days of hospital stay	3 (SD : 1)	
**4**	Median time of drain removal (days)	13 (range: 10-16)	
**5**	Malignancy detected in patients	2 (seminoma and mature teratoma)	
